# Gut Microbiota Composition Changes in Constipated Women of Reproductive Age

**DOI:** 10.3389/fcimb.2020.557515

**Published:** 2021-01-21

**Authors:** Hongxia Li, Jianwei Chen, Xiaojing Ren, Chuanli Yang, Shuai Liu, Xinshu Bai, Shuhua Shan, Xiushan Dong

**Affiliations:** ^1^ Department of General Surgery, Bethune Hospital Afﬁliated to Shanxi Medical University, Taiyuan, China; ^2^ Key Laboratory of Chemical Biology and Molecular Engineering of National Ministry of Education, Institute of Biotechnology, Shanxi University, Taiyuan, China

**Keywords:** chronic constipation, women of reproductive age, gut microbiota, 16S rRNA gene sequencing, influence factors

## Abstract

**Background:**

Chronic constipation is one of the most prevalent functional gastrointestinal disorders, yet its etiology is multifactorial, and the pathophysiological mechanism is still unclear. Previous studies have shown that the gut microbiota of constipated patients differs from healthy controls; however, many discrepancies exist in the findings, and no clear link has been confirmed between chronic constipation and changes in the gut microbiota. Growing evidence indicates that age, gender, and hormone levels can affect the composition of gut microbiota. The aim of this study is to examine the overall changes in gut microbiota within a specific sub-population of patients, namely, constipated women of reproductive age.

**Methods:**

We carried out a cross-sectional study comparing the fecal microbial composition of 30 healthy women and 29 constipated women using 16S rRNA gene sequencing. Only women of reproductive age were recruited to reduce the effects of age, gender, and hormone levels on the microbiome, and to prevent conflating the impact of these factors with the effects of constipation.

**Results:**

There were obvious differences in the gut microbiota in constipated women of reproductive age compared with the healthy controls, manifesting mainly as a significant increase in the abundance of *Bacteroides* (p < 0.05) and a significant decrease in the abundance of Proteobacteria (p < 0.01). The overall composition of the gut microbiota in each group was different, which was reflected in the ratios of Firmicutes to Bacteroidetes (F/B), which was 1.52 in the constipated group vs. 2.21 in the healthy group. Additionally, there was a significant decrease in butyrate-producing bacteria, like *Roseburia* and *Fusicatenibacter* (p < 0.01).

**Conclusion:**

The overall composition of the gut microbiota changed in constipated women of reproductive age, characterized by a loss in Proteobacteria and an increase in Bacteroidetes. Furthermore, the abundance of some butyrate-producing bacteria also reduced. These changes may reflect the unique interactions between host and some bacteria, or some bacterial metabolic products, which may be important targets for future studies to explore the pathogenesis of constipation.

## Introduction

Chronic constipation is a common symptom-based gastrointestinal disease without organic lesions. Its prevalence ranges from 2.6% to 26.9% in the general population, with a median value of 16% in all adults (Mugie and Benninga, 2011; Schmidt, 2014), and disproportionately affecting older adults and women. Chronic constipation often has a striking negative effect on quality of life (Belsey et al., 2010) and has been associated with a reduction in work productivity (Sun et al., 2011). Constipation is not only harmful to the intestinal tract itself but also can affect other diseases, and is known to aggravate cardiovascular and cerebrovascular diseases, leading to mental disorders (Li et al., 2016). Since it is not a life-threatening disease, and it is not a medical emergency, chronic constipation is often overlooked and not reported by patients suffering from it.

Chronic constipation is a multifactorial gastrointestinal disease, and its pathophysiological mechanism is still unclear. Constipation has previously been studied in the field of intestinal function, and only recently have new studies revealed that the gut microbiota of constipated patients differs from that of healthy controls. Some previous studies suggested there is a relationship between constipation and some altered abundance of certain species in the fecal microbiome using culture-based methods (Zoppi et al., 1998; Khalif et al., 2005). New advances in molecular biology methods have replaced traditional culture-based methods and are the current standard approach to analyze gut microbiota. These tools have revealed the tremendous diversity, richness, and functional capacity of human microbiome. Zhu et al. (2014) used 16S rRNA gene sequencing to prove that children with FC (functional constipation, FC) had a significantly decrease of Bacteroidetes, especially Prevotella, and a significant increase of some subgenera of Firmicutes, such as Lactobacillus. – used qPCR to determine that adults with FC had a significant loss in the abundance of Bifidobacterium and *Bacteroides*. Parthasarathy et al. (2016) used 16S rRNA metagenomics analysis to demonstrate that female patients suffering from chronic constipation or IBS-C (irritable bowel syndrome-constipation, IBS-C) had a significantly higher level of Bacteroidetes in their colonic mucosa.

These inconsistent findings are hard to explain. However, growing evidence indicates that age, sex, and hormone levels can affect the composition of intestinal flora (Bennett et al., 2016). Cross-sectional studies of fecal samples from adult individuals in various age groups suggest that there are age-related changes in gut microbiota composition and diversity. Longitudinal analyses indicate that the intestinal microbial population of healthy individuals is relatively stable for decades (Claesson et al., 2011). However, old age is associated with a more diverse and variable gut microbiome (Faith et al., 2013). With aging, the equilibrium state between intestinal flora and host worsens and gradually reaches a stage of dysbiosis (Kim, 2018; Riaz Rajoka et al., 2018; Xu and Zhu, 2019). In addition, some studies have shown an interaction between the microbiome and the endocrine system. Some bacteria can produce hormones and regulate the host’s hormonal homeostasis by inhibiting gene transcription. Similarly, host hormones may affect bacterial gene expression and bacterial growth and have consequences on host physiology (Rizzetto et al., 2018). The sex of the host also influences the gut microbiome and affects disease susceptibility, and these differences are the result of the actions of sex hormones (Ma, 2019). Importantly, all of these factors known to affect the gut microbiota are also known to influence susceptibility to constipation (Houghton et al., 2016; Lu and Velasco-Benítez, 2017; Shin et al., 2019).

It is conceivable that the large number of gut microbiota residing in the intestinal tract influences intestinal function. Therefore, in this study we examine the overall structure of gut microbiota in constipated women of reproductive age to reduce the effects of age, sex, and hormone levels. We aim to provide a clearer understanding of the role of the microbiome and to facilitate further research on the relationship between chronic constipation and gut microbiota.

## Materials and Methods

### Human Subjects

All participants were assessed using defecation related scales and a dietary habits questionnaire. Most of them were recruited from among the female staff of reproductive age of Shanxi Bethune Hospital. The constipated group needed to meet the Rome IV diagnosis standards of constipation and the normal group had a healthy lifestyle without any symptoms of constipation. All had a normal body mass index (BMI), indexes ranging from 18 to 25. All participants voluntarily enrolled and signed informed consent forms. The exclusion criteria for the study are summarized as follows: no children, men, pregnant women, or postmenopausal women; no patients with metabolic diseases, neuropsychiatric diseases, cancer, or such conditions as diabetes or Parkinson’s disease; they must have never undergone intestinal surgery; they must not have taken laxatives, antibiotics, probiotics, prebiotics, non-steroidal antiinflammatory drugs (NSAIDs), opioid drugs, traditional Chinese medicines, proton pump inhibitors (PPIs), or histamine receptor antagonists in the month preceding sample collection. All the fecal samples were processed on ice, stored in the Eppendorf tubes, and then transferred to a –80°C freezer within 30 min of collection. The study was approved by the ethics committee of Shanxi Bethune Hospital (Certificate No. XYLL-2019-124).

### Detection Methods

#### Genomic DNA Extraction and PCR Amplification

Microbial DNA of the samples was extracted using SDS (sodium dodecyl sulfate, SDS) method and then purity and concentration of DNA were assessed using agarose gel electrophoresis. An appropriate amount of sample DNA was taken in a centrifuge tube, and the sample was diluted to 1 ng/1g with sterile water. Using the diluted genomic DNA as the template, the V3–V4 regions of the bacterial 16S ribosomal RNA gene were amplified by PCR using the linker primer sequence CCTAYGGGRBGCASCAG, GGACTACNNGGGTATCTAAT, where the barcode is a twelve-base sequence unique to each sample. To ensure the efficiency and accuracy of amplification, Phusion high-fidelity PCR Master Mix with GC Buffer from New England Biolabs was used for PCR.

#### PCR Amplicon Purification and NovaSeq Sequencing

According to the concentration of duplicate PCR amplicons, the samples were mixed in equal amounts. Barcoded amplicons were recovered using gel recovery kits. TruSeq DNA PCR-free Sample Preparation Kit was used to construct the library. The constructed library was quantified using Qubit and q-PCR. After the library was established, Illumina NovaSeq6000 was used for computer sequencing.

### Information Analysis

#### Sequencing Data Processing

There are a certain proportion of dirty data among the raw data obtained from sequencing. In order to render the results of information analysis more accurate and reliable, the original data were first spliced and filtered to provide clean data. Then chimera filtering was conducted to obtain effective tags for subsequent analysis.

#### OTU Clustering and Species Annotation

In order to study the species composition of each sample, effective tags from all samples were clustered by OTUs (operational taxonomic units) with 97% identity using Uparse v7.0.1001 (http://www.drive5.com/uparse/).The sequence with the highest frequency of OTUs was selected as the representative sequence of OTUs. According to the OTU clustering results, species annotation was made for the representative sequence of each OTU to obtain the corresponding species information and species-based abundance distribution by using the Mothur method and SSUrRNA(sigmasubunit rRNA) database of SILVA132 (http://www.arb-silva.de/) and by using MUSCLE (version 3.8.31, http://www.drive5.com/muscle/) software for multiple sequence alignment.

#### Advanced Analysis

The smallest amount of data in the samples was taken as the standard for homogenization of all samples. Based on the homogenized data, the subsequent alpha diversity analysis was performed to find species richness and evenness information. We used Qiime software (version 1.9.1) to calculate Chao1 and Shannon indices; we used R software (version 2.15.3) to draw dilution and Rank abundance curves, all of which were for Alpha diversity index analysis of difference between the groups. Beta diversity analysis was performed to indicate the relative abundance distribution and significant differences of the gut microbiota between the groups. Metastats analysis used R software to perform permutation tests among groups under Phylum Class Order Family Species to determine *P* values. LEfSe software was used for LEfSe analysis, and the default screening value of LDA score was set to 4, which can be used for comparison of two or more groups. It emphasizes statistical significance and biological correlation, and it can be used to identify statistically different biomarkers between groups. Finally, we used Tax4Fun to predict the functional and metabolic capabilities of microbial communities. Tax4Fun is an open-source R package that predicts the functional or metabolic capabilities of microbial communities based on 16S rRNA datasets. The function of the microbial communities was predicted by constructing a linear relationship between the SILVA (from Latin *silva*, meaning forest, an improved data processing and web-based tool) classification and the KEGG (Kyoto Encyclopedia of Genes and Genomes) database.

## Results

### Species Richness and Diversity

**Figure 1:**
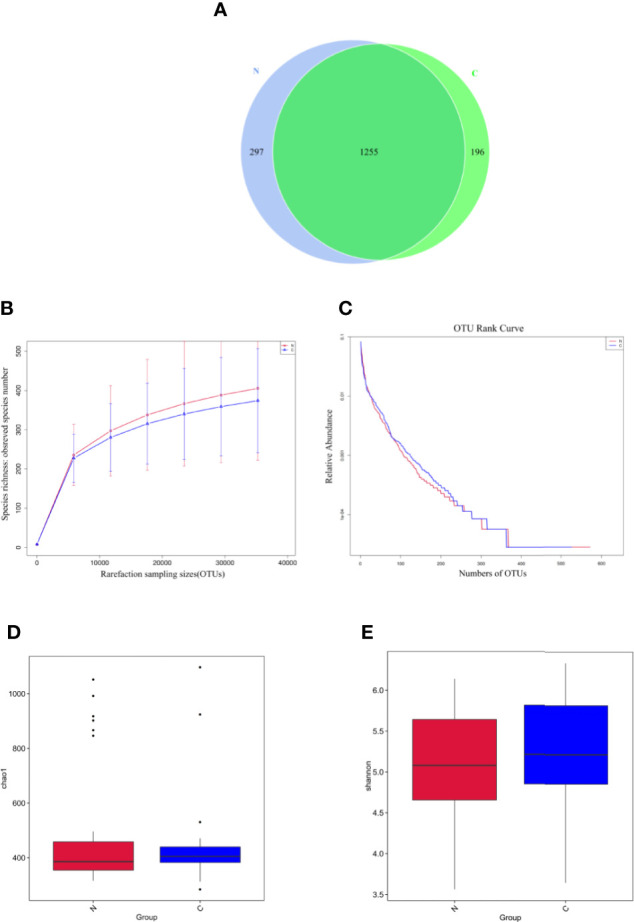
Species richness and diversity of the gut microbiome in two groups. N refers to the normal group; C refers to the constipated group. **(A)** Species richness and evenness based on the Venn diagram. The species richness in the constipated group is lower than that in the healthy group. **(B)** The rarefaction curve can directly reflect the rationality of sequencing data and indirectly reflect the richness of species in two groups. **(C)** The rank abundance curve reflects the richness and evenness of the species in two groups. **(D)** The Chao1 plot reflects species richness. **(E)** The Shannon plot reflects species diversity.

The fecal microbiomes of 29 constipated women of reproductive age and 30 healthy controls were analyzed by 16S rRNA gene sequencing, because one patient with constipation failed to collect stool sample. The characteristics of different group subjects are summarized in **Table 1**. There were no significant differences in age, body mass index, alanine transaminase, aspertate aminotransferase, fasting blood glucose, total bilirubin, albumin, total bile acid, cholesterol, triglyceride, high density lipoprotein, and low density lipoprotein of the two groups. A total of 7,414,293 sequencing reads were obtained from the 59 samples. To assess the alpha diversities of fecal flora in each group, five metrics were calculated: Venn diagrams, rarefaction curve, rank abundance curves, Shannon plots, and Chao1 plots, showing species enrichment and distribution. According to the OTU results and research requirements obtained by clustering, the common and unique OTUs among the groups were analyzed and displayed in a Venn diagram. The two groups had 1,255 common OTUs, and the 196 unique OTUs in the constipated group were lower than 297 in the control group (**Figure 1A**), indicating that the species diversity in the constipated group was reduced. The rarefaction curve for the healthy group was slightly higher than the constipated group, reflecting the richness in the healthy group. When the curve tended to be flat, it indicates that the sequencing data volume was considered reasonable. The slope of the rank abundance curve reflects the richness and evenness of the species in the sample. The richness of the species is reflected by the width of the curve. In the vertical direction, the smoothness of the curve reflects the uniformity of species in the sample. However, no significant difference was found in either the Shannon or Chao1 index (p > 0.05 for both), suggesting that the community richness and diversity of the two groups were approximately the same.

**Table 1:** Characteristics of the study groups.

Characteristics	Constipated (± S)	Control (± S)	*t*	*P*
Sex	Female	Female		
Age	33.63 ± 6.584	32.07 ± 6.968	-0.686	0.496
BMI	21.82 ± 2.863	22.25 ± 2.010	0.895	0.374
ALT	14.573 ± 6.939	15.377 ± 6.156	-0.474	0.637
AST	20.443 ± 7.875	21.000 ± 7.400	-0.282	0.779
FBG	4.916 ± 0.551	4.910 ± 0.308	0.052	0.959
TBIL	11.170 ± 4.284	12.070 ± 3.776	-0.863	0.392
ALB	45.000 ± 6.497	44.293 ± 2.049	0.568	0.572
TBA	2.717 ± 1.405	2.700 ± 1.634	0.042	0.966
CHO	4.244 ± 0.763	3.969 ± 0.699	1.458	0.150
TG	1.149 ± 1.040	0.935 ± 0.470	1.026	0.309
HDL	1.413 ± 0.272	1.430 ± 0.324	-0.220	0.826
LDL	2.410 ± 0.528	2.220 ± 0.466	1.475	0.146

### Relative Abundance Distributions at Different Levels of the Gut Microbiome in Two Groups

**Figure 2:**
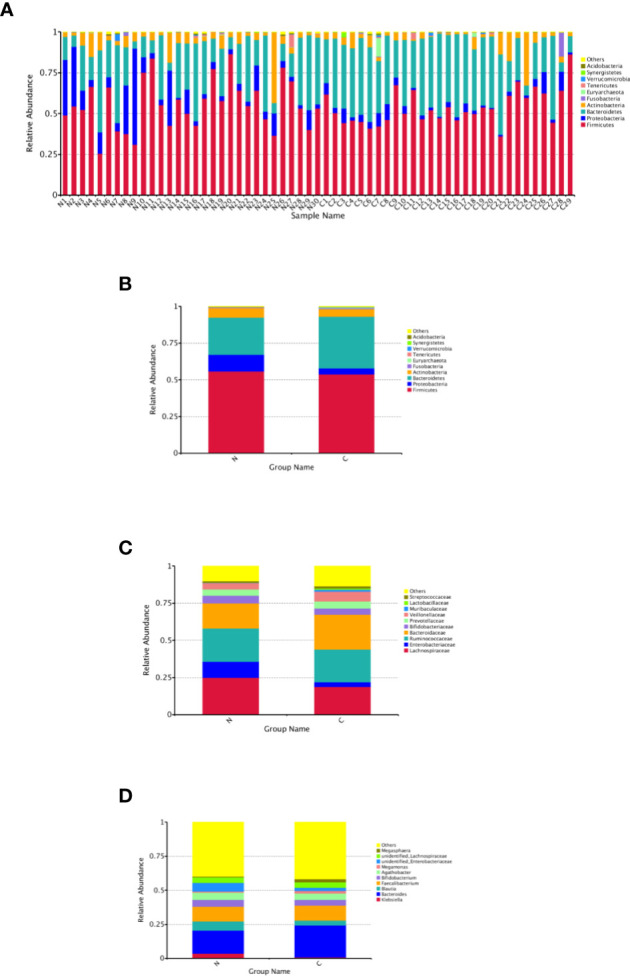
Relative abundance distribution at different levels of the gut microbiome in two groups. Here we analyze mainly at the level of Phylum, Family, Genus. **(A)** The relative abundance histogram of phyla in each sample. **(B)** The predominant phyla in the two groups. **(C)** The predominant family in the two groups. **(D)** The predominant genera in the two groups.

The relative abundance distribution at different levels of the gut microbiome in the two groups summarized in **Table 2**, which reflects the dominant flora and their relative proportion. The overall microbial composition at the phylum level in each sample is shown in **Figure 2A**, and the group data are shown in **Figure 2B**. A total of 25 bacterial phyla were detected in the gut microbiome of the two groups, including four fundamental phyla (Firmicutes, Bacteroidetes, Proteobacteria, and Actinobacteria), and other minor phyla. Firmicutes was the most prominent phylum, taking up 53.49 and 55.76%, respectively, of the gut microbiota in constipated women of reproductive age and healthy control group. The major families were Lachnospiraceae, Ruminococcaceae, Veillonellaceae, Peptostreptococcaceae, Lactobacillaceae, and Streptococcaceae. Bacteroidetes was the second major phyla in both groups, with a proportion of 35.14 and 25.22% in the two groups, including Bacteroidaceae, Prevotellaceae, Rikenellaceae, and Tannerellaceae. Proteobacteria, mostly Enterobacteriaceae, constituted the third most abundant phylum in the healthy control group, with a proportion of 11.49%, and the fourth most abundant phylum in the constipated group, with a proportion of 4.00%. Actinobacteria, mainly containing Bifidobacteriaceae, was the fourth most abundant phylum in the healthy control group and the third most abundant phylum in the constipated group (5.11 vs. 6.41%). According to the above analysis, there was a smaller ratio of Firmicutes/Bacteroidetes (F/B) in the constipated group than that in the healthy control group (1.52 vs. 2.21).

**Table 2:** The proportion and significant differences in the gut microbiome at different levels in the normal and constipated groups.

Taxa	Normal group	Constipated group	*p v*alue
**P_Firmicutes**	0.5576	0.5394	0.5774
c_Clostridia	0.4979	0.4294	0.0779
o_Clostridiales	0.4979	0.4294	0.0769
f_Lachnospiraceae g_Blautia g_Agathobacter g_Roseburia g_Fusicatenibacter g_unidentified_Lachnospiraceae	0.2499 0.0682 0.0529 0.0682 0.0079 0.0402	0.1873 0.0371 0.4679 0.0371 0.0129 0.0418	0.0659 0.1469 0.7253 0.0010^**^ 0.0489^*^ 0.9280
f_Ruminococcaceae g_Faecalibacterium g_unidentified_Ruminococcaceae	0.2192 0.1097 0.0360	0.2250 0.1067 0.0445	0.8122 0.9071 0.3007
f_Peptostreptococcaceae g_Romboutsia	0.0122 0.0102	0.0139 0.0116	0.7652 0.8091
c_Negativicutes	0.0422	0.0712	0.1578
o_Selenomonadales	0.0422	0.0712	0.1468
f_Veillonellaceae g_Megamonas g_Megasphaera g_Dialister	0.0414 0.0078 0.0065 0.0255	0.0665 0.0190 0.0213 0.0254	0.2238 0.4975 0.2268 0.9930
c_Bacilli	0.0104	0.0303	0.0619
o_Lactobacillales	0.0102	0.0301	0.0599
f_Lactobacillaceae g_Lactobacillus	0.0006 0.0006	0.0086 0.0086	0.0449^*^ 0.0529
f_Streptococcaceae g_Streptococcus	0.0086 0.0087	0.0154 0.0153	0.3257 0.3216
**P_Bacteroidetes**	0.2522	0.3514	0.0160^*^
c_Bacteroidia	0.2522	0.3514	0.0110^*^
o_Bacteroidales	0.2521	0.3513	0.0199^*^
f_Bacteroidaceae g_Bacteroides	0.1691 0.1691	0.2346 0.2346	0.0410^*^ 0.0450^*^
f_Prevotellaceae g_Paraprevotella g_unidentified_Prevotellaceae	0.0439 0.0021 0.0058	0.0484 0.0118 0.0030	0.8611 0.0639 0.8352
f_Muribaculaceae	0.0024	0.0127	0.3237
f_Rikenellaceae g_Alistipes	0.0178 0.0178	0.0210 0.0210	0.6503 0.6264
f_Tannerellaceae g_Parabacteroides	0.0105 0.0105	0.0234 0.0234	0.0050^*^ 0.0090^**^
**P_Proteobacteria**	0.1149	0.0400	0.0040^**^
c_Gammaproteobacteria	0.1121	0.0370	0.0040^**^
o_Enterobacteriales	0.1060	0.0324	0.0060^**^
f_Enterobacteriaceae g_Klebsiella g_Citrobacter g_unidentified_Enterobacteriaceae	0.1060 0.0357 0.0016 0.0638	0.0324 0.0077 0.0599 0.0226	0.0030^**^ 0.1528 0.0150^*^ 0.0220^*^
**P_Actinobacteria**	0.0641	0.0511	0.5005
c_unidentified_Actinobacteria	0.0526	0.0420	0.5754
o_Bifidobacteriales	0.0516	0.0414	0.6164
f_Bifidobacteriaceae g_Bifidobacterium	0.0516 0.0516	0.0414 0.0414	0.5954 0.5884

The normal group shows the average abundance of gut microbiota in healthy control subjects, n＝30. The constipated group shows the average abundance of gut microbiota in constipated women of reproductive age, n＝29. p values calculated from t test or Mann-Whitney U test. Prefixes p_, c_, o_, f_, and g_ refer to phylum, class, order, family, and genus. *p < 0.05; **p < 0.01.

At the family and the genus level, the top 10 most highly abundant bacteria in the healthy control group and constipated women of reproductive age were identifed. As shown in **Figures 2C, D**, these was mainly included Lachnospiraceae (with a proportion of 24.99 and 18.73%), Ruminococcaceae (21.92 vs. 22.50%), Bacteroidaceae (with a proportion of 16.91 and 23.46%), and Enterobacteriaceae (with a proportion of 10.60 and 3.24%) at the family level in two groups respectively, and mainly consisted of Bacteroidetes (16.91 vs. 23.46%), Feacalibacterium (10.67 vs. 10.97%), Bifidobacterium (5.16 vs. 4.14%), Blautia (6.82 vs. 3.71%), Agathobacter (5.29 vs. 4.68%), and Klebsiella (3.57 vs. 0.77%) at the genus level in two groups, respectively.

### Significant Differences in the Microbiota of Constipated Women of Reproductive Age and Healthy Controls

**Figure 3:**
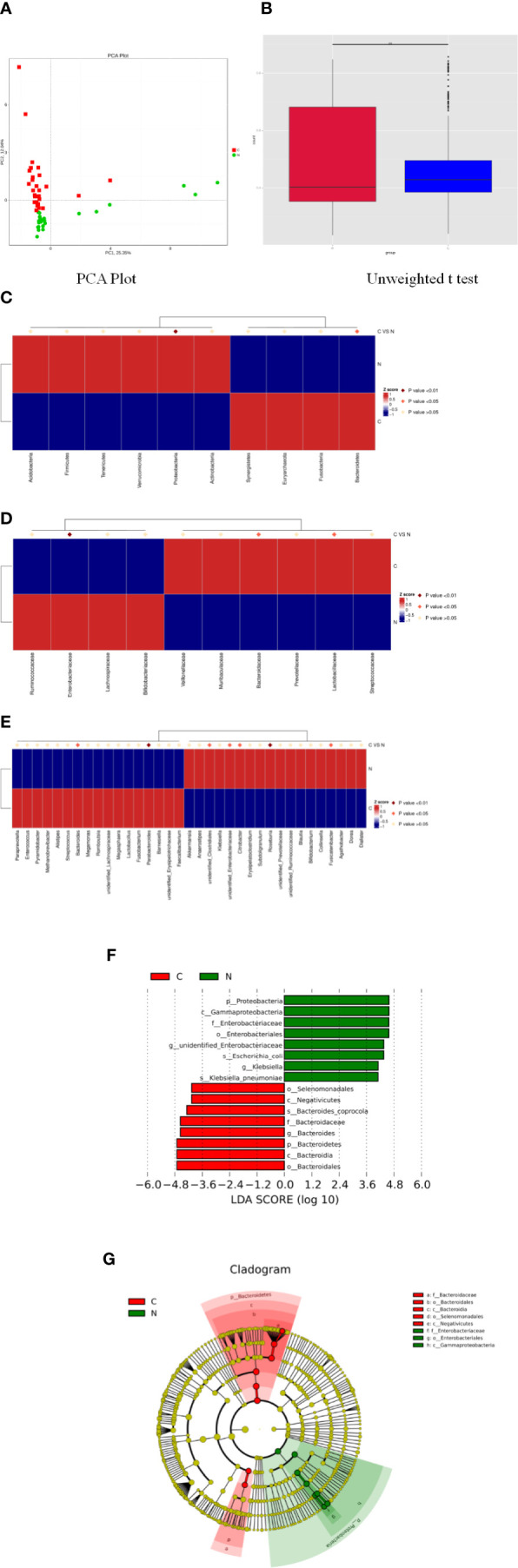
Significant differences in fecal microbiota between constipated women of reproductive age and healthy control subjects. **(A)** Principal component analysis of the two groups. **(B)** Box plots of beta diversity. **(C)** Comparison of the top 10 phyla. **(D)** Comparison of the top 10 families. **(E)** Comparison of the top 35 genus. **(F)** Distribution of LDA values displayed as a histogram, with an LDA value of >4 indicating statistical significance between the two groups. **(G)** Phylogenetic distribution of the microbiome of the two groups.

Principal component analysis indicated a remarkable separation in the fecal microbial composition between constipated women of reproductive age and healthy controls, with the first two principal component scores of PC1 (25.35%) and PC2 (12.04%) of explained variance (**Figure 3A**). The box diagram revealed that there was a significant difference in beta diversity between the two groups as determined by an unweighted t test (**Figure 3B**, p < 0.01).

The p values were obtained by hypothesis testing of species abundance data from different levels using the MetaStat method. A p-value of less than 0.05 or 0.01 was used to select the top 10 species. The data are shown in **Table 2** and are displayed as a heatmap with annotations in **Figure 3**. At the phylum level, Bacteroidetes was higher in the constipated group than that in the healthy control group (p < 0.05); nevertheless, Proteobacteria was lower in the constipated group than that in the healthy control group (p < 0.01). No differences were found in the other top 10 phyla of the two groups (**Figure 3C**). At the family level, the heatmap showed a significant increase in Bacteroidaceae and Lactobacillaceae (p < 0.05), and a significant decrease in Enterobacteriaceae (p < 0.01). The level of Lachnospiraceae (p = 0.0659) and Prevotellaceae (p = 0.0639) showed marginal values. However, there were no significant differences between the two groups for some common bacteria, such as Ruminococcaceae, Peptostreptococcaceae, Veillonellaceae, and Bifidobacteriaceae (**Figure 3D**). At the genus level, the top 35 most highly abundant bacteria were selected, as shown in **Figure 3E**. *Bacteroides* (p < 0.05) and *Parabacteroides* (p < 0.01) were more abundant in the constipated group; *Fusicatenibacter* (p < 0.05), *Citrobacter* (p < 0.05), and *Roseburia* (p < 0.01) were less abundant in the constipated group than those in the healthy group.

Using linear discriminant analysis coupled with effect size measurements, we confirmed that the phylum Bacteroidetes, the class Bacteroidia, the order Bacteroidales, the family Bacteroidaceae, and the genus *Bacteroides* were higher in the gut microbiota in constipated women of reproductive age, while the phylum Proteobacteria, the class Gammaproteobacteria, the order Enterobacteriales, the family Enterobacteriaceae, and the genus unidentified Enterobacteriaceae were higher in the gut microbiota in healthy controls (**Figure 3F**). The phylogenetic distribution of the microbiome of the two groups show a good correlation from phylum to genus level, suggesting that *Bacteroides* and Proteobacteria may be the crucial players involved in the pathogenesis of chronic constipation in women of reproductive age (**Figure 3G**).

### Functional Predictions for the Gut Microbiome in Constipated Women of Reproductive Age and Healthy Controls

**Figure 4:**
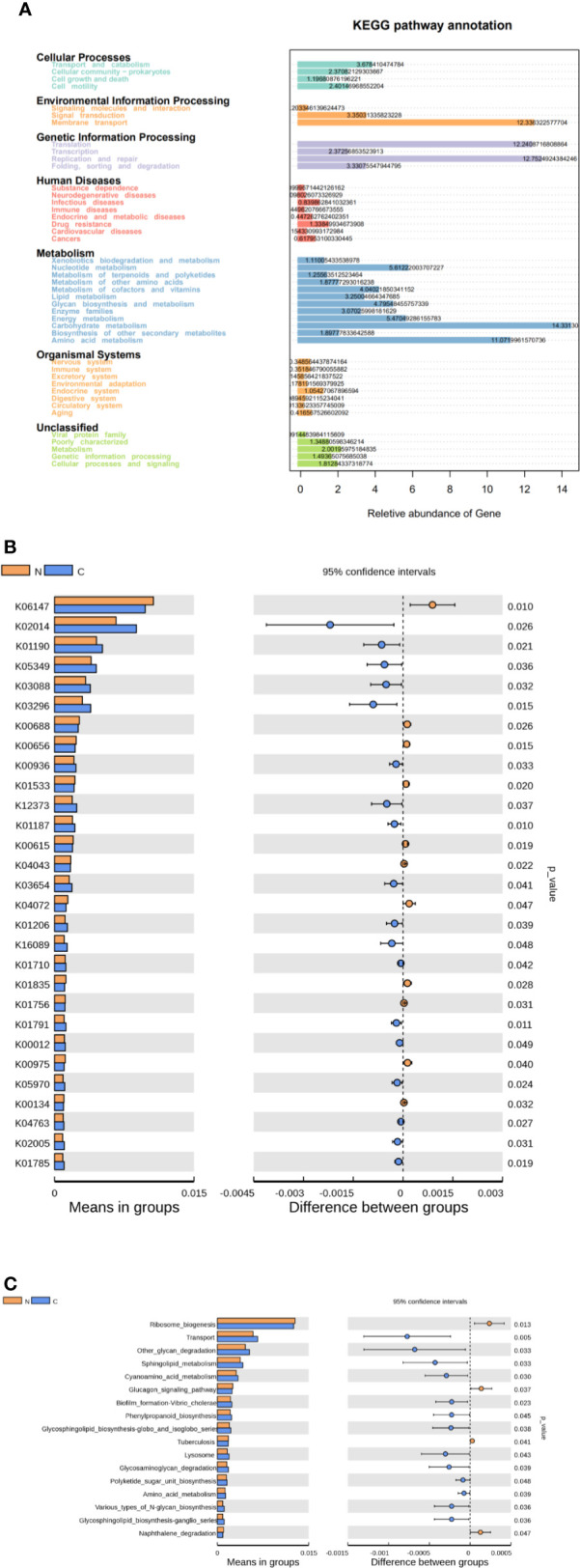
Functional predictions for the fecal microbiome in constipated women of reproductive age and healthy normal controls. **(A)** The relative abundance of gene in the KEGG pathway. **(B)** The KOs in the fecal microbiome of the two groups were shown to have significant differences (p < 0.05), which were identified using Tax4Fun. **(C)** The significant differences (p < 0.05) in KEGG pathways at level 3 for the fecal microbiome in constipated women of reproductive age and healthy normal individuals.

We used Tax4Fun to predict the functional and metabolic capabilities of microbial communities by constructing a linear relationship between the SILVA classification and the KEGG database. In the KEGG pathway annotations, the relative abundance of microbial genes in both groups was high in carbohydrate metabolism, amino acid metabolism, membrane transport of environmental information processing, translation and replication, and repair of genetic information process (**Figure 4A**). There were 28 KOs (KEGG Orthologues) be confirmed with significant differences in the fecal microbiomes between the two groups (p < 0.05; **Figure 4B**). Based on level 3 of KEGG pathway analyses, sphingolipid metabolism, cyanoamino acid metabolism, amino acid metabolism and biotin metabolism, transport, phenylpropanoid biosynthesis, glycosphingolipid biosynthesis, polyketide sugar unit biosynthesis, and various types of N-glycan biosynthesis were more abundance in fecal microbiome of the constipated group. The microbial gene functions involved ribosome biogenesis, glucagon signaling pathways, and naphthalene degradation were more abundant in the gut microbiome of the healthy normal control group (p < 0.05; see **Figure 4C**).

## Discussion

In this cross-sectional study, we compared fecal microbiota from constipated women of reproductive age and healthy controls, based on 16S rRNA sequencing to characterize the overall microbial differences. In contrast to previous studies, which enrolled men and women and a wide range of ages, from 18 to 80 years, we only included women of child-bearing age in our study to reduce the effects of gender, age, and hormone levels. We find that the ecological diversity and richness in the fecal microbiome in constipated women of reproductive age were similar to those in the healthy control individuals, whereas the differences at all taxonomic levels of the fecal microbiome between the two groups were significant, indicating that constipation is associated with an altered microbiome in the gut. As a whole, the fecal microbiome of constipated women of reproductive age exhibited an increased level of Bacteroidetes and decreased level of Proteobacteria, which was mostly explained by the increased abundance in the genus *Bacteroides* and the reduced numbers of Enterobacteriales. Significantly decreased levels of Proteobacteria and increased levels of Bacteroidetes resulted in a reduced ratio of F/B (Firmicutes to Bacteroidetes). Furthermore, the abundance of some butyrate-producing bacteria was also reduced.

The human colon contains a diverse microbial community, which is inhabited by hundreds of distinct species. Of these, 25% are *Bacteroides* (Eckburg et al., 2005). *Bacteroides* are strictly anaerobic, Gram-negative, non-motile, rod-shaped, and non-spore-forming bacteria, and comprises more than 92 species (Smith et al., 2006; Mancabelli et al., 2017). *Bacteroides*, the predominant genus within the human gut microbiota, usually plays a crucial role in degradation and fermentation of organic matter in the colon and are beneficial symbionts with their hosts (Salyers, 1995). Beneficial symbiosis requires the bacteria to sense changes in the environment so that they can adapt to alterations in their surroundings. The abundance of *Bacteroides* increased by 40% compared to that of healthy controls in this study, indicating that the increase in *Bacteroides* numbers may be based on the host’s constipated gut environment. The increased relative abundance of certain taxa in the presence of a gut disorder may not reflect a taxa-specific role in pathogenesis but may be linked to a global alteration of the gut microbiota’s homeostasis (Freitas et al., 2003). However, *Bacteroides* may simply need to turn on certain genes to change from friendly commensal to dangerous threat (Wexler, 2007). The best support is that *Bacteroides* also associated with infections, such as colitis and pouchitis (Shepherd et al., 1989; Kmiot et al., 1993), which suggests that *Bacteroides* may affect mucosal structure. Therefore, the increase of Bacteroidetes may also indicate an increase in pathogenic bacteria. What role Bacteroidetes plays in the constipated women of childbearing age requires further research and exploration.

Interestingly, we found a significant decrease in the abundance of the phylum Proteobacteria, in which Enterobacteriaceae was reduced. This result is similar to a previously published study that found that Enterobacteriaceae increased after constipated mice were given irritant laxatives (Takayama et al., 2019), revealing an association between high levels of the family Enterobacteriaceae and purgative activity. The family Enterobacteriaceae includes diarrheal pathogens such as Shigella and Salmonella. Furthermore, *Citrobacter rodentium* was found to be reduced in our study, and this bacterium belongs to the family Enterobacteriaceae, a close relative of the human diarrheal pathogen enterohemorrhagic and enteropathogenic *Escherichia coli* (Tsai et al., 2017; Zhu et al., 2018). Despite the fact that these diarrheal pathogens were not detected in our study, we still observed a reduction in *E. coli* in the constipated group as determined in the linear discriminant analysis. We assumed that an unclassified genus of the family Enterobacteriaceae might include similar bacteria, the reduction of which is associated with the pathogenesis of constipation. Consistent with the *in vitro* motility studies using human colon specimen, *E. coli* strain Nissle 1917 has been reported to promote gastrointestinal motility and muscle cell contractility (Bär et al., 2009).

Additionally, *Fusicatenibacter* and *Roseburia* were reduced in constipated women of reproductive age, and these are well-known butyrate-producing bacteria of the Lachnospiraceae family in the Firmicutes phylum. Butyrate producers are an abundant and phylogenetically diverse group of bacteria that are likely to play an important role in maintaining gut health, primarily through the production of butyrate. Studies have shown that the higher the abundance of butyrate-producing bacteria, the faster the colonic transit as a result of the influence of butyrate on gastrointestinal dynamics (Chassard et al., 2012). The butyrate produced by bacteria may accelerate colonic motility by stimulating the release of serotonin or promoting cholinergic pathways (Soret et al., 2010; Reigstad et al., 2015). A recent study shows that an abundance of butyrate-producing gut bacteria relieves constipation symptoms *via* short-chain fatty acids production and hormone secretion (Zhuang et al., 2019). However, we found no changes in the bacteria of Butyrate-metabolic-capability in the Tax4Fun analysis. This suggests that we need to look at the role of butyric acid-producing bacteria in the future using metabolomics.

The data of functional analysis suggest that the alteration of pathways involved in metabolism, biosynthesis, genetic information process, and environmental information processing is associated with chronic constipation. The 16S rRNA gene sequencing method has its own disadvantages as it could not identify microorganisms at the species level or the strain level or provide direct data on the crucial changes in the functionality of the microbiota (Aßhauer et al., 2015). Future studies should include samples from men and women of various ages, ethnic origins, geographical regions, and dietary differences, and apply metagenomics sequencing analysis techniques to explore the changes in the function of gut microbial genes.

## Conclusion

Overall, we find evidence for gut microbiota dysbiosis in constipated women of reproductive age by discussing the fecal microbiota compositional shifts in case groups as compared to healthy controls. The variations may predict the unique interactions between hosts and some certain bacteria, or some specific bacterial metabolic products, which may aid future exploration of the pathogenesis of constipation. Narrowing this study to a small sub-population in order to avoid the influences of age, sex, and hormonal differences had a clear impact on the meaningfulness of the data and made data interpretation more straightforward.

Notably, the taxonomic characters of microorganisms extracted from fecal samples included both the indigenous flora as well as the allochthonous microbiota and thus may not be completely representative of the resident gut microbial population. Nevertheless, analysis of fecal flora is also a promising method for a rapid screen with the aim of identifying biomarkers associated with chronic constipation, since the collection of fecal samples is noninvasive and no special clinical procedures are required. The limitations of the present study should be considered. We did not detect structural changes in colonic mucosal flora, nor did we perform shotgun metagenome analysis to understand intestinal flora function. We did not perform metabolomics analyses to explore the pathogenesis of constipation at a molecular level. Our study preliminarily identified some potential microflora, which still requires further validation based on clinical samples and animal models. Elucidating the differences in the fecal microbiome of female constipated patients of reproductive age may provide the foundation to improve our understanding of the pathogenesis of chronic constipation in specific populations and could support the development of novel therapeutic options aimed at modifying the gut microbiota.

## Data Availability Statement

The original contributions presented in the study are publicly available. This data can be found here: https://www.ncbi.nlm.nih.gov/. SRA accession: PRJNA636012.

## Ethics Statement

The studies involving human participants were reviewed and approved by the ethics committee of Shanxi Bethune Hospital. The patients/participants provided their written informed consent to participate in this study.

## Author Contributions

XD coordinated the project and conceived of the study. HL and JC recruited patients and conducted the clinical trials. XR and CY participated in its design and coordination. SL and XB collected clinical samples. SS helped refine the design of the clinical trials and helped interpret the data. HL performed the statistical analysis, and prepared and revised the manuscript. All authors had read and approved the final manuscript.

## Funding

This research was supported by the Natural Science Foundation of Applied Basic Research of Shanxi Province, China (201801D121199) and the Research Project of Shanxi Provincial Health and Family Planning Commission, China (2018019).

## Conflict of Interest

The authors declare that the research was conducted in the absence of any commercial or financial relationships that could be construed as a potential or actual conflict of interest.

## References

[B38] Aßhauer K. P. Wemheuer B. Daniel R. (2015). Tax4Fun: predicting functional profiles from metagenomic 16S rRNA data. Bioinformatics 31, 2882–2884. 10.1093/bioinformatics/btv287 25957349 PMC4547618

[B33] Bär F. Von Koschitzky H. Roblick U. Bruch HP Schulze L. Sonnenborn U. (2009). Cell-free supernatants of Escherichia coli Nissle 1917 modulate human colonic motility: evidence from an in vitro organ bath study. Neurogastroenterol. Motil. 21, 559–566. 10.1111/j.1365-2982.2008.01258.x 19220758

[B3] Belsey J. Greenfield S. Candy D. (2010). Systematic review: impact of constipation on quality of life in adults and children. Aliment. Pharmacol. Ther. 31, 938–949. 10.1111/j.1365-2036.2010.04273.x 20180788

[B11] Bennett G. Malone M. Sauther M. L. Cuozzo F. P. White B. Nelson K. E. (2016). Host age, social group, and habitat type influence the gut microbiota of wild ring-tailed lemurs (Lemur catta). Am. J. Primatol. 78, 883–892. 10.1002/ajp.22555 27177345

[B34] Chassard C. Dapoigny M. Scott K. P. Crouzet L. Del'homme C. Marquet P. (2012). Functional dysbiosis within the gut microbiota of patients with constipated-irritable bowel syndrome. Aliment. Pharmacol. Ther. 35, 828–838. 10.1111/j.1365-2036.2012.05007.x 22315951

[B12] Claesson M. J. Cusack S. O’Sullivan O. Greene-Diniz R. de Weerd H. Flannery E. (2011). Composition, variability, and temporal stability of the intestinal microbiota of the elderly. Proc. Natl. Acad. Sci. U.S.A. 108 Suppl 1, 4586–4591. 10.1073/pnas.1000097107 20571116 PMC3063589

[B22] Eckburg P. B. Bik E. M. Bernstein C. N. Purdom E. Dethlefsen L. Sargent M. (2005). Diversity of the human intestinal microbial flora. Science 308, 1635–1638. 10.1126/science.1110591 15831718 PMC1395357

[B13] Faith J. J. Guruge J. L. Charbonneau M. Subramanian S. Seedorf H. Goodman A. L. (2013). The long-term stability of the human gut microbiota. Science 341, 1237439. 10.1126/science.1237439 23828941 PMC3791589

[B26] Freitas M. Tavan E. Cayuela C. Diop L. Sapin C. Trugnan G. (2003). Host-pathogens cross-talk. Indigenous bacteria and probiotics also play the game. Biol. Cell. 95, 503–506. 10.1016/j.biolcel.2003.08.004 14630386

[B20] Houghton L. A. Heitkemper M. Crowell M. Emmanuel A. Halpert A. McRoberts J. A. (2016). Age, Gender and Women’s Health and the Patient. Gastroenterology 150, 1332–1343. 10.1053/j.gastro.2016.02.017 27144622

[B7] Khalif I. L. Quigley E. M. Konovitch E. A. (2005). Alterations in the colonic flora and intestinal permeability and evidence of immune activation in chronic constipation. Dig. Liver Dis. 37, 838–849. 10.1016/j.dld.2005.06.008 16169298

[B9] Kim S. E. Choi S. C. Park K. S. Park M. I. Shin J. E. Lee T. H. (2015). Change of fecal flora and effectiveness of the short-term VSL3 Probiotic treatment in patients with functional constipation. J. Neurogastroenterol. Motil. 21, 111–120. 10.5056/jnm14048 25537674 PMC4288088

[B14] Kim S. (2018). The Gut Microbiota and Healthy Aging: A Mini-Review. Gerontology 64, 513–520. 10.1159/000490615 30025401 PMC6191326

[B28] Kmiot W. A. Youngs D. Tudor R. Thompson H. Keighley M. R (1993). Mucosal morphology, cell proliferation and faecal bacteriology in acute pouchitis. Br. J. Surg. 80 (11), 1445–1449. 10.1002/bjs.1800801132 8252361

[B5] Li X. Feng R. Wu H. Zhang L. Zhao L. Dai N. (2016). Psychological characteristics and GoNogo research of patients with functional constipation. Medicine Baltimore 95, e5685. 10.1097/MD.0000000000005685 28033259 PMC5207555

[B19] Lu P. L. Velasco-Benítez C. A. (2017). Sex, Age, and Prevalence of Pediatric Irritable Bowel Syndrome and Constipation in Colombia: A Population-based Study. J. Pediatr. Gastroenterol. Nutr. 64, e137–e141. 10.1097/MPG.0000000000001391 27579696

[B18] Ma Z. S. (2019). How and Why Men and Women Differ in Their Microbiomes: Medical Ecology and Network Analyses of the Microgenderome. Adv. Sci. (Weinh.) 6, 1902054. 10.1002/advs.201902054 31832327 PMC6891928

[B24] Mancabelli L. Milani C. Lugli G. A. Turroni F. Mangifesta M. Viappiani A. (2017). Unveiling the gut microbiota composition and functionality associated with constipation through metagenomic analyses. Sci. Rep. 7, 9879. 10.1038/s41598-017-10663-w 28852182 PMC5575163

[B2] Mugie S. M. Benninga M. A. (2011). Epidemiology of constipation in children and adults: a systematic review. Best Pract. Res. Clin. Gastroenterol. 25, 3–18. 10.1016/j.bpg.2010.12.010 21382575

[B10] Parthasarathy G. Chen J. Chen X. Wolf P. G. Gaskins H. R. Bharucha A. E. (2016). Relationship Between Microbiota of the Colonic Mucosa vs Feces and Symptoms, Colonic Transit, and Methane Production in Female Patients With Chronic Constipation. Gastroenterology 150, 367–379. 10.1053/j.gastro.2015.10.005 26460205 PMC4727996

[B35] Reigstad C. S. Salmonson C. E. Rainey J. F. Szurszewski J. H. Linden D. R. Sonnenburg J. L. (2015). Gut microbes promote colonic serotonin production through an effect of short-chain fatty acids on enterochromaffin cells. FASEB J. 29, 1395–1403. 10.1096/fj.14-259598 25550456 PMC4396604

[B16] Riaz Rajoka M. S. Zhao H. Li N. Lu Y. Lian Z. Shao D. (2018). Origination, change, and modulation of geriatric disease-related gut microbiota during life. Appl. Microbiol. Biotechnol. 102, 8275–8289. 10.1007/s00253-018-9264-2 30066188

[B17] Rizzetto L. Fava F. Tuohy K. M. (2018). Connecting the immune system, systemic chronic inflammation and the gut microbiome: The role of sex. J. Autoimmun. 92, 12–34. 10.1016/j.jaut.2018.05.008 29861127

[B25] Salyers A. A. (1995). “Fermentation of polysaccharides by human colonic anaerobes,” in Dietary Fibre. Eds. Cherbut C. Barry J. L. Lairon D. Durand M. (Paris: John Libbey Eurotext Press), 29–35.

[B1] Schmidt F. M. (2014). Prevalence of constipation in the general adult population: an integrative review. J. Wound Ostomy Continence Nurs. 41, 70–76. 10.1097/01.WON.0000438019.21229.b7 24378694

[B29] Shepherd N. A. Hultén L. Tytgat G. N. Nicholls R. J. Nasmyth D. G. Hill M. J. (1989). Pouchitis. Int. J. Colorectal Dis. 4 (4), 205–229. 10.1007/BF01644986 2693561

[B21] Shin A. Preidis G. A. Shulman R. (2019). The Gut Microbiome in Adult and Pediatric Functional Gastrointestinal Disorders. Clin. Gastroenterol. Hepatol. 17, 256–274. 10.1016/j.cgh.2018.08.054 30153517 PMC6314902

[B23] Smith C. J. Rocha E. R. Paster B. J. (2006). “The medically important Bacteroides spp. in health and disease,” in The Prokaryotes: A Handbook on the Biology of Bacteria. Ed. Dworkin M. (New York: Springer Press), 381–427. 10.1007/0-387-30747-8_14

[B36] Soret R. Chevalier J. De Coppet P. Poupeau G. Derkinderen P. Segain J. P. (2010). Short-chain fatty acids regulate the enteric neurons and control gastrointestinal motility in rats. Gastroenterology 138, 1772–1782. 10.1053/j.gastro.2010.01.053 20152836

[B4] Sun S. X. Dibonaventura M. Purayidathil F. W. Wagner J. S. Dabbous O. Mody R. (2011). Impact of chronic constipation on health-related quality of life, work productivity, and healthcare resource use: an analysis of the National Health and Wellness Survey. Dig. Dis. Sci. 56, 2688–2695. 10.1007/s10620-011-1639-5 21380761

[B30] Takayama K. Takahara C. Tabuchi N. (2019). Daiokanzoto (Da-Huang-Gan-Cao-Tang) is an effective laxative in gut microbiota associated with constipation. Sci. Rep. 9, 3833. 10.1038/s41598-019-40278-2 30846728 PMC6405880

[B32] Tsai P. Y. Zhang B. He W. Q. Zha J. M. Odenwald M. A. Singh G. (2017). IL-22 Upregulates Epithelial Claudin-2 to Drive Diarrhea and Enteric Pathogen Clearance. Cell Host Microbe 21, 671–681. 10.1016/j.chom.2017.05.009 28618266 PMC5541253

[B27] Wexler H. M. (2007). Bacteroides: the good, the bad, and the nitty-gritty. Clin. Microbiol. Rev. 20 (4), 593–621. 10.1128/CMR.00008-07 17934076 PMC2176045

[B15] Xu C. Zhu H. (2019). Aging progression of human gut microbiota. BMC Microbiol. 19, 236. 10.1186/s12866-019-1616-2 31660868 PMC6819604

[B8] Zhu L. Liu W. Alkhouri R. Baker R. D. Bard J. E. Quigley E. M. (2014). Structural changes in the gut microbiome of constipated patients. Physiol. Genomics 46, 679–686. 10.1152/physiolgenomics.00082.2014 25073603

[B31] Zhu Q. Dupont C. L. Jones M. B. Pham K. M. Jiang Z. D. DuPont H. L. (2018). Visualization-assisted binning of metagenome assemblies reveals potential new pathogenic profiles in idiopathic travelers’ diarrhea. Microbiome 6, 201. 10.1186/s40168-018-0579-0 30409177 PMC6225641

[B37] Zhuang M. Shang W. Ma Q. Strappe P. Zhou Z. (2019). Abundance of Probiotics and Butyrate-Production Microbiome Manages Constipation via Short-Chain Fatty Acids Production and Hormones Secretion. Mol. Nutr. Food Res. 63, e1801187. 10.1002/mnfr.201801187 31556210

[B6] Zoppi G. Cinquetti M. Luciano A. Benini A. Muner A. Bertazzoni Minelli E (1998). The intestinal ecosystem in chronic functional constipation. Acta Paediatr. 87, 836–841. 10.1111/j.1651-2227.1998.tb01547.x 9736230

